# Supplementary material for: Identification of Sources and Transformations of Nitrate in the Intense Human Activity Region of North China Using a Multi-Isotope and Bayesian Model
Source: Int J Environ Res Public Health. 2021 Aug 16;18(16):8642. doi: 10.3390/ijerph18168642 (PMC8392111; doi:10.3390/ijerph18168642)
Supplement: Supplementary file 1 [file ijerph-18-08642-s001.zip › ijerph-1295402-supplementary.pdf]

Supplementary figure caption
Supplementary figure 1 Hydrogeological cross-section of Ye River basin
Supplementary figure 2 Variations of the concentration and isotopic compositions for nitrate in the Ye River.

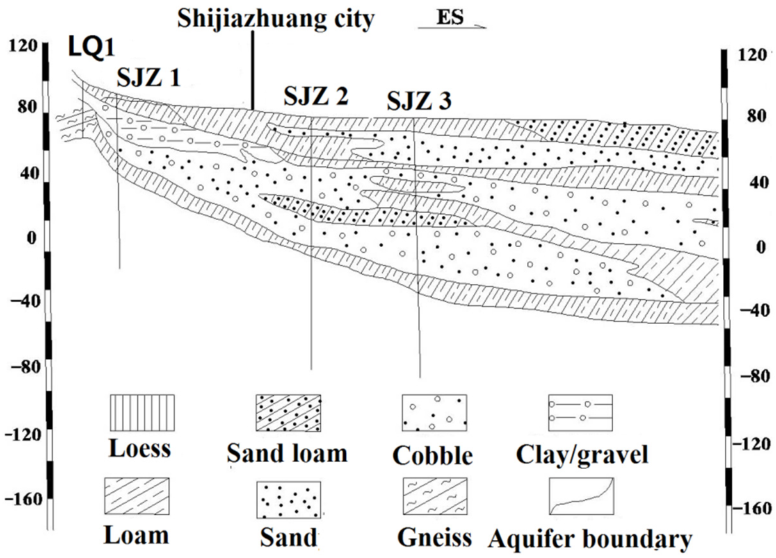

Supplementary Figure S1 Hydrogeological cross-section of Ye River basin

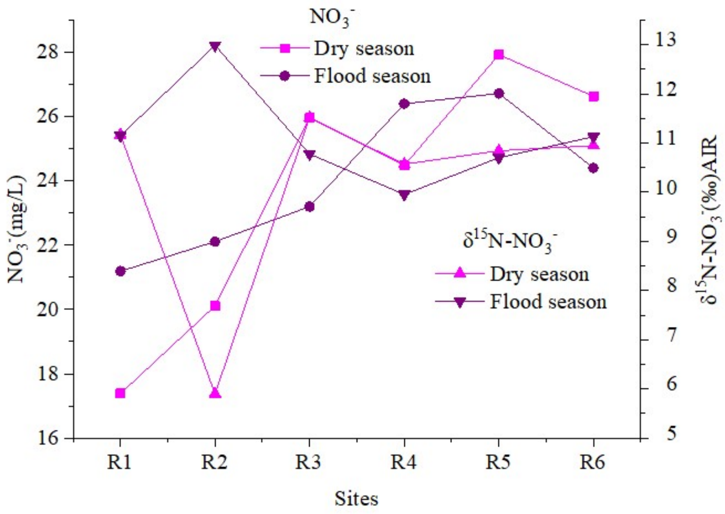

Supplementary Figure S2 Variations of the concentration and isotopic compositions for nitrate in the Ye River.
